# Iterative Adaptations in a Physical Activity Program for Children with Autism: A Feasibility and Implementation Study

**DOI:** 10.3390/healthcare14111502

**Published:** 2026-05-28

**Authors:** Miriam Richter, Marie K. Taylor, Teresa Lindstedt, Annika Lundkvist Josenby, Olof Rask, Christine T. Ekdahl

**Affiliations:** 1 Division of Clinical Neurophysiology, Department of Clinical Sciences, Lund University, SE-221 84 Lund, Sweden; miriam.richter@med.lu.se (M.R.); marie.taylor@med.lu.se (M.K.T.); 2 Division of Child and Adolescent Psychiatry, Department of Clinical Sciences, Lund University, SE-221 84 Lund, Sweden; olof.rask@med.lu.se; 3 Clinical Neurophysiology, Department of Medical Imaging and Physiology, Skåne University Hospital, SE-221 85 Lund, Sweden; 4 Epilepsy Center, Department of Clinical Sciences, Lund University, SE-221 85 Lund, Sweden; 5 Division of Pediatrics, Skåne University Hospital, SE-221 84 Lund, Sweden; teresa.lindstedt@skane.se (T.L.); annika.lundkvist@med.lu.se (A.L.J.); 6 Department of Health Sciences, Lund University, SE-221 85 Lund, Sweden

**Keywords:** autism spectrum disorder, physical activity, feasibility study, implementation, adapted physical activity

## Abstract

**Background:** Children and adolescents with autism spectrum disorder (ASD) are generally more sedentary and engage less in physical activity (PA) than their peers. Despite increasing evidence of benefits, practical guidance on implementing adapted PA programs in real-world settings remains limited. This study aimed to document iterative adaptations, implementation processes, and participant experiences in a structured PA intervention for children with ASD. **Methods:** Nineteen children aged 10–14 years with ASD participated in an adapted PA intervention delivered across three cohorts. The program was progressively modified based on observed barriers and participant feedback. Post-intervention conversations with participants and/or parents were used to assess feasibility and experiences. **Results:** Most participants trained on-site in small groups and were predominantly boys, many with comorbid ADHD/ADD. Baseline engagement in organized PA was low. Adaptations included adjustments to session structure, group size, instructor-to-participant ratio, and activity content to enhance predictability and autonomy. Individual tailoring and flexible delivery were essential to accommodate neurodevelopmental diversity and fluctuating motivation. Post-intervention feedback suggested generally positive acceptability, while findings should be interpreted descriptively. **Conclusions:** This study provides preliminary, practice-based insights into how structured PA programs may be iteratively adapted for children with ASD in a supportive clinical context. The findings highlight practical considerations for implementing adapted PA in clinical and community settings.

## 1. Introduction

Children and adolescents with autism spectrum disorder (ASD) often experience barriers to participation in physical activity (PA), despite well-documented benefits for physical and mental health. ASD is a neurodevelopmental condition characterized by differences in social communication and interaction, as well as restricted and repetitive behaviors and interests, often accompanied by variations in sensory responses, affecting about 1% of the global population [1,2,3]. These characteristics may influence participation in structured PA contexts.

Children and adolescents with ASD are generally more sedentary and engage less frequently in moderate-to-vigorous PA than typically developing (TD) peers [4,5,6,7,8,9,10]. Participation is low in both organized and non-organized PA, particularly in team sports, unstructured peer activities, and commercial gym settings [11]. In addition, children with ASD report lower enjoyment of physical activity, with greater symptom severity associated with reduced enjoyment of physical, social, and organized activities [12,13]. In children with ASD, motor impairments, social difficulties, reduced cognitive flexibility, and female sex are associated with lower physical activity levels [14,15,16]. Reduced activity contributes to an increased risk of overweight and obesity [17,18,19] and is linked to adverse metabolic outcomes [7,20]. Differences in activity are evident from early childhood and tend to widen with age [6,21,22]. Together with environmental and social barriers, these findings highlight the need for targeted interventions.

Structured PA interventions may benefit children and adolescents with ASD across multiple domains [23,24,25], including physical fitness [26,27], motor skills [28,29,30], social and communication abilities [31,32,33,34], reductions in stereotypic behaviors [35,36], executive functions [37,38,39], and mental health outcomes such as anxiety and depression [40,41]. Existing research has described a range of PA interventions for youth with ASD [42]. However, much of the current evidence is derived from controlled research settings with small samples, which may limit generalizability.

Children with ASD without intellectual disability often face barriers to organized and adaptive programs due to challenges concerning predictability, sensory sensitivities, motor coordination, and social demands [43]. Research has identified intrapersonal, interpersonal, and environmental barriers [44]. Parents frequently report the need for supervision, behavioral challenges, limited access to suitable programs, and logistical constraints [45], while adolescents describe participation as conditional upon perceived competence, confidence, motivation, predictability, adjustment to demands, and autonomy [46]. Despite growing awareness of these factors, there remains limited real-world detail on how adapted PA programs are modified during delivery in clinical and community settings.

The primary aim of this exploratory feasibility and implementation study was to document iterative adaptations in coaching strategies, session structure, and logistical organization in a structured PA program for children with ASD across three consecutive cohorts. A secondary aim was to describe participant- and parent-reported experiences of the intervention. The study was not designed to evaluate effectiveness, and no prespecified outcome measures of intervention effects were applied.

## 2. Methods

### 2.1. Study Design

This study was designed as an exploratory, descriptive feasibility and implementation study aimed at documenting practical adaptations required to deliver a structured PA program for children with ASD in a real-world clinical setting. The study was not hypothesis-driven and did not include a control group. The intervention was delivered across three consecutive cohorts, with iterative modifications introduced between cohorts based on observed challenges and participant feedback. The study was not intended to evaluate intervention effectiveness, and all analyses were descriptive.

### 2.2. Participants and Recruitment

Participants were identified through administrative systems at the Child and Adolescent Psychiatric and Habilitation Clinics in southern Sweden (Malmö, Lund) from January 2020 through December 2022. Eligible families were contacted by telephone and provided with child-adapted information; criteria were reviewed, and those who agreed were invited to participate. Inclusion criteria were a confirmed ASD diagnosis corresponding to DSM-5 levels 1–2 (American Psychiatric Association, 2022) [1], age 10–14 years, and ability to communicate verbally. Exclusion criteria included moderate-to-severe intellectual disability (American Psychiatric Association, 2022) [1], recent traumatic brain injury, active systemic autoimmune disease, or neurodegenerative disorders. Data on demographics, clinical characteristics, sleep habits, and ongoing pharmacological treatments were obtained from routine clinical records and parent-reported information. Written informed consent was obtained from legal guardians. Participants were enrolled in three consecutive cohorts. At baseline, parent-reported information on PA levels and previous experience with organized training was collected.

Beyond the present study, participants were invited to take part in additional related studies, including blood sampling for immune markers [47] and an exploratory pre–post study involving parent questionnaires, cognitive testing, and actigraphy [48]. Participation in these additional components was voluntary, and not all participants contributed to all procedures. The procedures were conducted at time points before or after the physical activity intervention, depending on the specific study protocol.

### 2.3. Intervention

Participants took part in an 8–12-week PA program, with 1–3 sessions per week, delivered as supervised group training or home-based sessions with parental support to enhance feasibility and reduce COVID-19 infection risk. Home sessions mirrored group structure and intensity, with instructional videos; cohorts 2–3 received loaned equipment. Participants were encouraged to keep training diaries and to wear pulse watches, with a video tutorial provided to ensure correct use. Group sessions, led by Lund University physiotherapy students (instructor-to-participant ratio 1:1–4), were held at Friskis & Svettis or gymnasiums at Child and Adolescent Habilitation Clinics. The relatively high instructor-to-participant ratio was considered important for supporting engagement, individual adaptation, and overall feasibility. Each 45–60 min session included warm-up, circuit or ball-based activities, and relaxation, with verbal instructions and demonstrations (Appendix A). A detailed description of the exercise and relaxation components is provided in Supplementary Materials S1. Activities aimed to raise heart rate (HR) while emphasizing enjoyment; team sports and competitive elements were excluded. Instructors received training in autism, common comorbidities, low-arousal approaches, and seizure management. Researchers and instructors met regularly to monitor engagement and address challenges. At the end of each cohort, all participants were invited to a concluding session and received a diploma or medal. Public health recommendations related to COVID-19 were followed throughout the study.

### 2.4. Iterative Adaptation Process

The intervention included core structural elements, while content and delivery were adapted iteratively across cohorts. Across cohorts, session structure, instructional strategies, scheduling, group size, instructor-to-participant ratio, and relaxation components were progressively adapted in response to observed barriers and feedback from children, parents, and instructors. The present manuscript focuses on these practical adjustments. The intervention should therefore be understood as an evolving implementation process rather than a fixed or standardized program. To facilitate replicability, the intervention is here conceptualized as comprising core and adaptable components, i.e., adaptable elements that could be modified across cohorts.

Core Components Included:

- A predictable session structure and duration (45–60 min), including consistent phases (warm-up 10 min, aerobic training 25 min, and relaxation 10 min);
- Individualized pacing and adaptation;
- Supportive instructor–participant relationships, including a low-arousal approach;
- A relatively high instructor-to-participant ratio to support individualized guidance and engagement;
- Emphasis on non-competitive, enjoyment-focused, and pulse-raising activities.

Adaptable Components Included:

- Group size and instructor-to-participant ratio (within a consistently high-support framework across cohorts);
- Specific activity content (e.g., circuit vs. ball-based);
- Scheduling and session timing;
- Format and structure of relaxation activities.

The underlying intervention logic was that a structured but flexible physical activity format, combined with individualized support and opportunities for autonomy, would promote engagement and sustained participation among children with ASD in a real-world setting.

### 2.5. Data Collection and Analysis

Data sources included parent-reported baseline information on PA levels, as well as parent- and participant-reported feedback collected through semi-structured conversations at, or shortly after, the final training session. Additional data sources included instructor observations and reflections, training diaries, and attendance records. Conversations were conducted by the training instructors in a clinical setting and documented as written notes. They followed a semi-structured format covering recurring topic areas such as overall experience, perceived barriers and facilitators, motivation, and practical aspects of participation. No fixed or fully standardized conversation guide was used, and minor variations in questioning occurred across cohorts. Conversations typically lasted approximately 10–20 min. The study was not designed for inferential statistical analysis.

Pulse watches worn during PA sessions recorded HR, with the relative increase calculated as (average HR − minimum HR)/minimum HR × 100. HR data were used descriptively to indicate engagement in pulse-raising activity and were not intended as standardized physiological outcome measures.

Feasibility was operationalized descriptively based on attendance, retention, and the ability of participants to engage in and complete the intervention. Attendance was defined as the total number of completed sessions per participant. Retention was defined as continued participation beyond the initial two sessions of the intervention. Engagement was interpreted as the ability to participate in structured sessions as intended, based on instructor observations and attendance records. No predefined quantitative thresholds for adequate attendance or retention were applied, as the study was designed to reflect real-world variability in participation. All feasibility measures were therefore interpreted descriptively (Supplementary Table S1).

Acceptability was assessed based on overall participant- and parent-reported experiences collected through semi-structured conversations conducted at or shortly after the final training session. Reported experiences were categorized as positive, neutral, or negative. These categories were assigned by two members of the research team (MR and CTE), who independently reviewed the written notes and reached consensus through discussion. The categorization reflects an overall descriptive interpretation of reported experiences rather than a validated or standardized measure. The absence of a standardized interview guide limits comparability across cohorts and should be considered when interpreting the findings.

The descriptive material (conversation notes, observations, and diaries) was analyzed using an inductive descriptive approach inspired by thematic analysis. The analysis followed three steps: (1) familiarization with the material through repeated reading; (2) identification of recurrent patterns related to barriers, facilitators, and adaptations; and (3) discussion and refinement of interpretations within the research team. To enhance credibility, two researchers (MR and CTE) independently reviewed the material and reached consensus through discussion, and preliminary interpretations were discussed within the wider research group. No formal qualitative methodology (e.g., predefined coding framework, inter-rater reliability testing, or formal thematic analysis) was applied. The findings are therefore presented as descriptive implementation feedback rather than formally derived qualitative themes.

### 2.6. Ethical Considerations

The study was conducted in accordance with the Declaration of Helsinki and approved by the Swedish Ethical Review Authority (approval number 2020-03207) and registered at ClinicalTrials.gov (Protocol Record 2020-03207). Written informed consent was obtained from all legal guardians.

## 3. Results

### 3.1. Participant Characteristics and Baseline Physical Activity

Nineteen children with ASD participated across three cohorts (Table 1). The majority were boys (*n* = 14), and most had a comorbid ADHD/ADD diagnosis (*n* = 15). None had an intellectual disability. Four children reported sleep disturbances at baseline (*n* = 1 missing data), although most were treated with melatonin (*n* = 14). Three participants had no medication.

Parent-reported baseline PA indicated low engagement in organized PA (Table 2). Twelve children were reported as currently never participating in organized PA, four participants had no prior experience of organized PA, and six did not attend school-based physical education. Most children engaged in approximately 50–99 min of weekly low-level PA in everyday activities, such as biking to school, walking the dog, or playing with friends during school breaks.

### 3.2. Attendance and Feasibility

All findings are reported descriptively based on observed or reported data, without inference regarding causality or effectiveness. They are presented without predefined thresholds, in line with the exploratory study design. Attendance varied substantially between participants, ranging from 7 to 28 on-site sessions during the intervention period (Table 3). Most participants trained on-site (*n* = 11), some with additional home-based training (*n* = 2), and some only at home (*n* = 3). Three children discontinued participation after one to two sessions due to health issues, adjustment difficulties, or low motivation. Despite variability in attendance, many children who remained in the program participated regularly throughout the intervention. One participant per cohort completed most on-site sessions individually, with a designated instructor in a separate room adjacent to the main training area. Families generally reported that on-site sessions were associated with better follow-through, whereas home-based training was primarily used as a complementary option when attendance was not feasible. This pattern was based on parent-reported feedback and attendance records rather than formal comparative analysis. Variation in attendance reflected real-world conditions and individual differences in participation rather than planned differences in intervention dose.

Pulse-watch data were available from a subset of participants (*n* = 9). The mean number of recorded training sessions per participant was 13.7 (range 4–23). Across participants, the mean relative increase in HR during training sessions was 64.28 ± 13.85%. Acceptance of pulse watches varied, with some children finding them motivating and others experiencing discomfort and declining to use them consistently. Technical complications also contributed to missing data. These data are presented as descriptive contextual information indicating engagement in pulse-elevating activity and were not collected using standardized protocols (e.g., %HRmax). Therefore, they cannot be compared with established exercise physiology thresholds or used to classify specific exercise intensity.

### 3.3. Iterative Adaptations Across Cohorts

**Figure 1:**
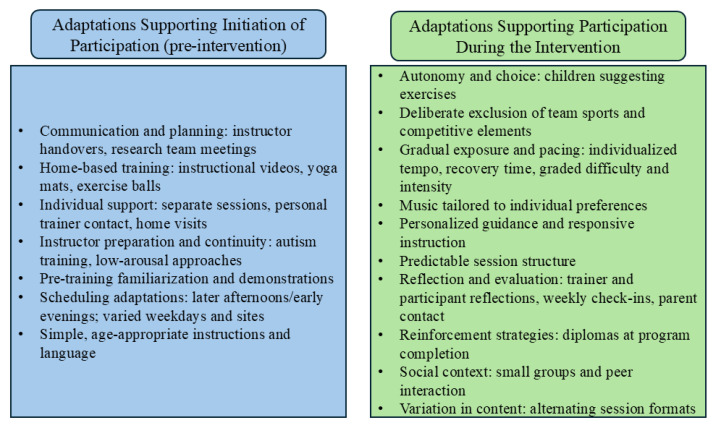
Descriptive overview of adaptations implemented across cohorts 1–3. The figure summarizes adaptations introduced pre- and during the intervention, based on recurring observations from instructor reflections, training diaries, and parent- and child-reported feedback. Adaptations are grouped according to their general function: supporting initiation of participation (**left** panel) and supporting continued participation during the intervention (**right** panel). The figure represents a descriptive summary of implementation-related observations and does not reflect the frequency, relative importance, or effectiveness of individual adaptations.

Progressive adaptations were implemented across cohorts in response to observed barriers and participant feedback (Table 4, Figure 1). Key domains of adaptation included scheduling and logistics, session structure and content, instructor-to-participant ratio, instructional strategies, and relaxation components. As the intervention evolved across cohorts, the following descriptions reflect sequential adaptations rather than directly comparable conditions.

#### 3.3.1. Cohort 1

Sessions included a warm-up (10 min), circuit training with three difficulty levels (25 min), and structured relaxation incorporating elements of basic body awareness therapy (10 min). Instructors trained together prior to meeting the children. Activities were tailored to each child’s abilities, with some opportunities for suggestions and modifications and possibilities to choose either early or late afternoon training. The intensity of PA was gradually increased to ensure sufficient cardiovascular load. Instructors reported that early repetition appeared to support predictability, while flexible PA difficulty, individualized pacing, varied content, and music tailored to preferences were used to support engagement. Weekly follow-ups and occasional home visits supported adherence. A video-recorded dance session was introduced as an alternative component of the home-based training. Reflections from children and instructors were documented throughout the cohort. Instructors contributed to a joint final report at the cohort’s conclusion.

#### 3.3.2. Cohort 2

A handover from Cohort 1 instructors ensured continuity. Building on previous feedback from parents and participants, group sizes were slightly reduced, the instructor-to-participant ratio was reduced, and PA programs were scheduled only in the late afternoon. PA sessions were alternated between circuit training and ball-based activities, with the aim of further increasing variation and engagement. Ball-based activities included games using small and large balls, performed individually or with peers, involving both upper and lower extremities. The relaxation component was simplified, incorporating age-appropriate language, yoga, and mindfulness. Additional adaptations included clearer visual demonstrations, self-selected body positioning during relaxation, prioritization of large balls and balance exercises, and gradual increases in strength demands. Instructors noted higher observable engagement during ball-based sessions compared to circuit training, and obstacle courses were found to be particularly motivating. The observations were based on instructor reflections, training diaries, and informal feedback from participants and were not derived from standardized quantitative measures.

#### 3.3.3. Cohort 3

Sessions continued to be scheduled for later afternoons, and group sizes and instructor-to-participant ratios were kept similar for feasibility and support, typically involving two instructors and up to four children. Training continued to combine ball-based and circuit activities, beginning with a warm-up and ending with relaxation, though the content became predominantly ball-based to match participant preferences. However, the content of the PA program in Cohort 3 was more flexibly adapted in real time to suggestions from the participants, with alternatives offered for more challenging tasks.

Overall, core components with adaptations during all three cohorts comprised a predictable session structure, individualized pacing, varied activities, opportunities for autonomy, supportive instructor relationships, and gradual exposure to physical challenges. These factors were identified through a descriptive review of observational notes, instructor reflections, and parent- and participant-reported feedback and are presented as descriptive implementation feedback rather than formal qualitative findings (Figure 1).

### 3.4. Acceptability of the PA Intervention

Most parents and children reported positive experiences, while a minority described neutral or negative experiences (Table 3). The categorization into positive, negative, or neutral reflects a simplified descriptive summary rather than a standardized measure. Positive acceptability was linked to familiarity with the training context, supportive instructor relationships, and opportunities to influence session content.

### 3.5. Reported Barriers and Facilitators for Participation

Several children initially experienced anxiety in the unfamiliar setting, which was generally perceived to decrease as predictability increased, according to parents and instructors. Motivation to participate was supported by opportunities for choice, individualized adaptations, and feeling acknowledged. PA preferences varied, with activities experienced as enjoyable or challenging depending on the child. Adaptations, including avoiding team sports or modifying exercises, were generally appreciated, though occasional rule changes were perceived as disruptive for some. Home-based training suited children who were highly uncomfortable with new environments or peers, whereas others reported greater motivation during on-site sessions. Over time, children in Cohorts 1–3 reported increased confidence, a sense of contribution, and an improved ability to perform exercises, particularly when able to influence session content. Overall, motivation and meaningfulness increased, with many expressing interest in continuing PA, although some anticipated challenges in new settings or with unfamiliar people. These findings are based on participant- and parent-reported experiences and instructor observations and are presented descriptively without inference regarding effectiveness. They reflect descriptive feedback derived from semi-structured conversations and observational material.

### 3.6. Practical and Social Context

Families reported logistical challenges, including transportation, scheduling, and limited parental energy, as well as disruptions to daily routines. Consistent instructors were valued, and peer presence could be perceived as both motivating and challenging, depending on the child. Some parents noted positive social outcomes, such as supportive relationships with instructors and peers, while others preferred minimal social demands. Home-based training was favored when families faced logistical constraints. These observations were derived from parent-reported feedback and are presented descriptively.

### 3.7. Reported Bodily Experiences and Well-Being During Participation

Children reported both physical challenges (e.g., overheating, shortness of breath, muscle pain) and benefits from the PA sessions, including increased energy, endurance, reduced movement-related discomfort, and greater mobility. Parents noted improvements in mood, energy, and daily activity, as well as enhanced body awareness, motivation, and understanding of PA benefits. For some families, the PA intervention became a shared health-promoting activity. A minority of participants experienced added stress, highlighting the need for flexible, individualized adaptations and support. These outcomes reflect perceived experiences reported by participants and parents rather than objectively measured changes.

## 4. Discussion

This exploratory feasibility and implementation study examined practical adaptations introduced to support participation in a structured PA program for children with ASD. Across three consecutive cohorts, training structure, coaching strategies, and logistics were refined iteratively based on observed barriers and participant feedback. The intervention evolved substantially across cohorts in terms of scheduling, content, group size, and instructor support; therefore, the cohorts should be understood as sequential adaptations rather than equivalent versions of a single intervention. This limits comparisons across cohorts and precludes conclusions regarding which specific components may have influenced engagement or acceptability. The distinction between core and adaptable components may nevertheless support future implementation in clinical and community settings by identifying essential elements while allowing contextual flexibility.

Overall, most children and parents reported positive experiences, although attendance varied, and a few participants discontinued. The findings should be interpreted as descriptive implementation feedback rather than evidence of intervention effects or systematically derived qualitative themes.

The majority of participants were boys. Research suggests that girls with ASD often engage in lower levels of physical activity than boys [49,50], though findings are mixed and sex differences require further investigation [5,11]. The underrepresentation of girls in the present sample limits conclusions regarding sex-specific needs and highlights an area for future research. As a clinical cohort, comorbidity was common, enhancing ecological validity. ADHD prevalence was somewhat higher than reported in other clinical samples [51]. Children with ADHD often have lower motor skills, aerobic fitness, and engagement in organized PA than TD peers [52,53]. The high level of comorbidity may have influenced both participation patterns and perceived barriers.

Heart-rate data were available for a subset of participants and are presented as descriptive contextual information. As the data were not collected using standardized protocols (e.g., %HRmax) [54], they cannot be compared with established exercise physiology thresholds or used to classify PA intensity.

The iterative adaptation process required ongoing reflection and flexibility from instructors. Barriers included anxiety, post-school fatigue, unfamiliarity with PA, distractibility, low endurance, frustration, difficulty following instructions, and physical discomfort. Children’s preferences varied, with some benefiting from structure and others from autonomy; relaxation exercises also posed challenges for some participants. Facilitators included varied activities (circuit training, ball games, dance), social interaction, perceived improvements, opportunities to influence training, and meaningful contribution through research. These factors were identified descriptively based on observations and reported experiences and should not be interpreted as systematically derived determinants. Practical constraints and routine disruptions were barriers, while motivation, interest, and achievement supported engagement. The close interaction between instructors and participants may also have influenced reported experiences.

Previous research highlights similar facilitators and barriers. Enjoyment and meaningfulness drive PA motivation in adolescents with ASD, further enhanced by supportive companions; social, task, and environmental demands can hinder participation [46]. Predictability, freedom of choice, and influence over activity are important. Parents facilitate PA through encouragement and logistics but report limited institutional support [55]. The present findings are consistent with these observations and extend them by illustrating how such factors may be addressed in practice through iterative adaptation. However, given the evolving nature of the intervention, the observations should not be interpreted as evidence of the relative effectiveness of specific program components.

Several alternative explanations for the reported positive experiences should be considered. High instructor-to-participant ratios and individualized attention may have contributed to engagement independently of specific program components. In addition, supportive relationships with instructors, increased familiarity with the training environment over time, and repeated contact within a structured research context may have influenced both participation and perceived acceptability. Other factors, such as novelty effects and end-of-intervention rewards (e.g., diplomas or medals), may also have contributed to positive experiences. These contextual elements are inherent to the study setting and make it difficult to disentangle the effects of specific adaptations from broader aspects of participation.

Consequently, the reported experiences cannot be attributed to individual components of the intervention but should instead be understood as descriptive accounts within a supportive and structured implementation context. Future studies using more controlled designs are needed to clarify the relative contribution of specific intervention components.

Michaud and Harvey [56] advocate shifting PA research and practice for children with ASD from deficit- to strength-based approaches, highlighting facilitators across six socio-ecological levels. The present study aligns with this perspective by emphasizing individualized adaptations, flexible delivery, and relational support. However, in contrast to some previous findings, competitive elements and team sports were not experienced as facilitators in this cohort. This discrepancy may reflect differences in participant characteristics and context.

Future interventions should build on the described barriers and facilitators to further explore which adaptations are most relevant for different subgroups of children and adolescents with ASD. More systematic evaluation approaches, including defined outcome measures and qualitative methodologies, are warranted.

### Strengths and Limitations

This work addresses a relevant and practical topic, focusing on the real-world implementation of physical activity programs for children with ASD. The iterative design across cohorts is a clear strength and reflects applied research conditions. Most participants in the study had low baseline activity, which is consistent with the target population. The majority completed the intervention in small groups and expressed interest in continuing PA, suggesting that the described adaptations were compatible with participant engagement and were well-suited to their needs.

A key limitation is the absence of a control group, which precludes any conclusions regarding intervention effects. The study was not designed to evaluate efficacy but to explore feasibility and implementation processes. Selection bias was unavoidable, as participation required time, flexibility, and active engagement from both children and families. Families who chose to participate in a structured physical activity program within a clinical research context may represent a subgroup with higher motivation, greater resources, and increased openness to health-related interventions than the broader ASD population. This may have contributed to an overestimation of both feasibility and acceptability. In addition, the findings may not be transferable to families facing greater logistical challenges, such as limited availability, higher levels of daily stress, transportation barriers, or lower baseline motivation for participation in structured activities.

Furthermore, the inclusion criteria required verbal communication and excluded individuals with moderate-to-severe intellectual disabilities. Consequently, the findings primarily reflect verbally fluent children with ASD without intellectual disability and may not be generalizable to the broader autism spectrum. In addition, the predominance of male participants with comorbid ADHD, along with recruitment constraints related to COVID-19 restrictions, small group sizes, and the underrepresentation of girls, further limits the generalizability of the results. Future studies should aim to include additional subgroups with autism and explore implementation strategies across a broader range of functional levels and contextual conditions.

Participation in concurrent studies represents an additional potential source of bias. Some participants were involved in multiple study components, including blood sampling, questionnaires, cognitive testing, and actigraphy, which may have increased both participant burden and contact frequency with the research team. This broader engagement may have influenced motivation, retention, and adherence to the intervention, as well as perceived acceptability. Increased familiarity with the research setting, repeated interactions with staff, and a higher level of overall study involvement may have contributed positively to participation and reported experiences. As such, feasibility and acceptability may partly reflect the supportive context of a multi-component research setting.

HR data were incomplete due to discomfort or technical issues, which limits the interpretation of exercise intensity. Furthermore, HR was not assessed using standardized protocols, and the results should therefore be interpreted as indicative rather than comparable to established physiological measures. The limited availability and variability of HR data should be considered when interpreting these findings.

The study relied on descriptive data sources, including observations, training diaries, and semi-structured conversations. No formal qualitative analysis (e.g., coding framework, thematic saturation, or inter-rater validation) was conducted, which limits the methodological rigor of the qualitative component. In addition, feasibility and acceptability were not defined using predefined criteria and were instead assessed descriptively based on attendance, retention, and reported experiences. Finally, as the intervention was iteratively adapted, experiences were assessed somewhat differently across cohorts. This limits comparability between cohorts and makes it difficult to distinguish between intervention components and contextual adaptations.

Future studies would benefit from more standardized evaluation procedures involving instructors, parents, and participants to improve understanding of program acceptability. In particular, clearly defined outcome measures, standardized feasibility criteria, and structured qualitative methodologies would strengthen future research in this area.

## 5. Conclusions

Children and adolescents with ASD are often less physically active than their peers and may have limited access to organized, adapted physical activity. In this exploratory feasibility and implementation study, a structured PA program was iteratively adapted to support participation in a clinical sample with low baseline physical activity and a high degree of ADHD comorbidity.

The findings provide preliminary, practice-based insights into how adapted PA programs may be implemented and adjusted in real-world clinical settings. They suggest that feasibility and acceptability may be achieved within a subgroup of verbally fluent children with ASD without intellectual disability participating in a supportive and structured research context.

However, given the exploratory design, small sample size, evolving intervention structure, selection bias, and descriptive outcome assessment, these findings should be interpreted with caution and should not be considered evidence of effectiveness.

Future studies are needed to evaluate implementation and outcomes using predefined feasibility and acceptability criteria, more stable intervention designs, and more rigorous qualitative and quantitative methodologies to better understand which components are most relevant for different subgroups.

## 6. Perspective

Within adapted physical activity, there is a growing emphasis on participation as a primary outcome, particularly for neurodivergent populations. The present study contributes practice-oriented insights by illustrating how structured physical activity programs can be dynamically adjusted to accommodate heterogeneity in motivation, sensory processing, social preferences, and daily functioning among children with autism spectrum disorder. Whereas much of the existing literature focuses on intervention effects, fewer reports detail the operational processes required for sustainable implementation. By documenting iterative modifications in session organization, instructor support, and environmental structuring, this study adds applied knowledge relevant to clinicians, adapted physical activity specialists, and community providers. The findings reinforce the importance of relational competence, contextual sensitivity, and flexible delivery models in promoting inclusive participation. As the field continues to move toward strength-based and person-centered approaches, such implementation-focused evidence may support the development of scalable models that bridge research and everyday practice.

## Figures and Tables

**Table 1:** Participant characteristics.

Variable	Participants
	*n* = 19
Age: median years (range)	11 (9–13)
Sex: boys % (*n*)	74 (14)
ADHD or ADD ^1^ % (*n*)	79 (15)
Other psychiatric diagnosis ^2^ % (*n*)	32 (6)
Somatic diagnosis ^3^ % (*n*)	26 (5)
Schooling % (*n*)	
-Regular school with no support	16 (3)
-Regular school with support	42 (8)
-Adapted school form	26 (5)
-Missing data	16 (3)
Medication % (*n*)	
-Melatonin	74 (14)
-Antipsychotic medication	10 (2)
-ADHD medication ^4^	63 (12)
-Antidepressants (SSRIs)	16 (3)
-Anti-seizure medication	16 (3)

^1^ *n* = 1 ADD, *n* = 14 ADHD; ^2^ anxiety disorder, obsessive–compulsive disorder, tic disorder, language disorder, depressive disorder, eating disorder, and enuresis; ^3^ generalized epilepsy, unilateral hearing loss, psoriasis, and migraine; ^4^ central stimulants, atomoxetine, and guanfacine.

**Table 2:** Baseline physical activity (PA).

Participant	Cohort	Reported Baseline Physical Activity		
		Current Organized PA	Weekly Low-Intensity PA (Minutes)	Previous Organized PA
1	1	No; does not participate in school-based PE	50–99	Yes
2		Yes, occasionally	50–99	Yes
3		No; does not participate in school-based PE	100–150	Yes
4		Yes, once or twice per week	>150	No
5		No	>150	No
6		No; does not participate in school-based PE	50–99	No
7		No	50–99	No
8		No; does not participate in school-based PE	50–99	Yes
9	2	No	50–99	Yes
10		Yes, ≥5 times per week	>150	No
11		Missing data	Missing data	Missing data
12		Yes, once or twice per week	100–150	No
13	3	No; but participates in school-based PE twice a week	>150	Yes
14		No; but participates in school-based PE twice a week	50–99	Yes
15		No; does not participate in school-based PE	50–99	No
16		No	100–150	Yes
17		No; does not participate in school-based PE	50–99	Yes
18		Yes, occasionally	50–99	Missing data
19		Yes, occasionally	100–150	No

Data on current and previous experience of organized PA and daily-life low-activity PA levels at baseline were collected via a questionnaire completed by the parents.

**Table 3:** Attendance and reported acceptability of the PA intervention.

Participant	Cohort	Attendance (Number of Sessions) and Training Settings	Reported Acceptability
1	1	11, at home	Negative
2		8, mainly at home	Neutral
3		24, on-site	Positive
4		14, on-site	Neutral
5		21, on-site	Positive
6		21, at home	Positive
7		21, at home	Neutral
8		Drop out (after 1–2 sessions)	Drop out
9	2	15, on-site	Positive
10		10, on-site	Positive
11		27, mainly on-site	Positive
12		17, on-site	Positive
13	3	7, on-site	Positive
14		28, on-site	Positive
15		Drop out (after 1–2 sessions)	Drop out
16		Drop out (after 1–2 sessions)	Drop out
17		15, on-site	Positive
18		18, on-site	Positive
19		13, on-site	Positive

Attendance data were derived from training diaries, attendance lists maintained by the instructors, and pulse watch recordings. Based on evaluations by the participants and/or their parents at the end of the intervention, responses were summarized as reflecting an overall positive, negative, or neutral experience of the intervention.

**Table 4:** Key intervention characteristics and cohort-specific adaptations of the PA intervention.

Core Components	Cohort 1	Cohort 2	Cohort 3
Child influence on content	Limited choice	Choice between predefined activities	High degree of child-led choice
Group size	1–6	1–4	1–4
Instructor–attendance ratio	1:1–4	1:1–2	1:1–3
Relaxation	Structured	Structured	Flexible
Scheduling	Early and late afternoon	Late afternoon	Late afternoon
Session structure	Circuit-based	Circuit mixed with ball-based	Mostly ball-based

Adaptations were introduced iteratively across cohorts based on feasibility observations and participant feedback.

## Data Availability

The data presented in this study are available on request from the corresponding author. The data are not publicly available due to privacy or ethical restrictions.

## Supplementary Materials

The following supporting information can be downloaded at: https://www.mdpi.com/article/10.3390/healthcare14111502/s1, Table S1: Operational definitions of feasibility and acceptability measures; Materials S1: Detailed description of physical activity program and relaxation exercises.

## Appendix A

### Summary of Intervention Components

The intervention included structured sessions consisting of three main components:

(1) Warm-up;
(2) Circuit-based and/or ball-based physical activities adapted to individual ability levels;
(3) Relaxation exercises incorporating elements of yoga and mindfulness.

Activities were adjusted to participant needs and preferences, with variations in content, intensity, and instructional strategies across cohorts.
