# Supplementary material for: Iterative Adaptations in a Physical Activity Program for Children with Autism: A Feasibility and Implementation Study
Source: Healthcare (Basel). 2026 May 28;14(11):1502. doi: 10.3390/healthcare14111502 (PMC13256154; doi:10.3390/healthcare14111502)
Supplement: Supplementary file 1 [file healthcare-14-01502-s001.zip › healthcare-4267496-supplementary.pdf]

Supplementary Table S1 Operational definitions of feasibility and acceptability measures

Domain	Definition	Data source	Interpretation
**Attendance**	Total number of completed sessions per participant	Attendance logs, training diaries	Descriptive (no predefined threshold)
**Retention**	Continued participation beyond first two sessions	Attendance records	Descriptive
**Engagement**	Ability to participate in structured sessions as intended	Instructor observations, attendance	Descriptive
**Acceptability**	Overall reported experience categorized as positive, neutral, or negative	Participant and parent conversations	Assigned by MR and CTE based on written notes

Supplementary Material S1: Detailed description of physical activity program and relaxation exercises

A) Circuit Training_Program with Three Difficulty Levels

TRAINING PROGRAM

Levels: Easy – Medium – Hard

EASY

1. Air Cycling

Lie on your back on a mat with your arms by your sides. Keep your legs in the air while extending and bending your knees as if you are cycling.

2. Squats (Not as Deep)

Stand with your feet hip-width apart. Keep your back straight with your gaze forward. Bend your knees as much as you are able. Push back up and return to the starting position. Make sure your heels stay on the floor and that your knees point in the same direction as your toes. Sets: ___ Reps: ___

3. Back Extension

Lie on your stomach with your hands just outside your shoulders. Look down at the floor. Lift your upper body off the floor and slowly lower back down.

4. Seated to Reach Up

Start in a standing position. Bend your knees and sit down. Stand back up in any way you prefer and stretch your arms overhead.

5. Quadruped Arm Lift

Stand on all fours without collapsing in the shoulders. Keep your head aligned with your upper body and gently tuck your chin. Engage your lower abdominals by drawing the navel toward the spine. Slowly lift one arm until it is in line with the body. Keep tension in the core throughout. Lower slowly and repeat on the other side. Sets: ___ Reps: ___

6. Running in Place

Stand with your feet parallel. Run in place. Roll through the foot and lift your heels toward your glutes.

7. Boxing

Stand with your feet about shoulder-width apart. Hold your arms in a boxing position in front of your body. Punch forward alternately with clenched fists.

8. Single-Leg Balance

Stand on one leg with slightly bent knees and an upright posture. Imagine swaying like a blade of grass in the wind. Maintain the position until the leg becomes tired. Increase difficulty by closing your eyes.

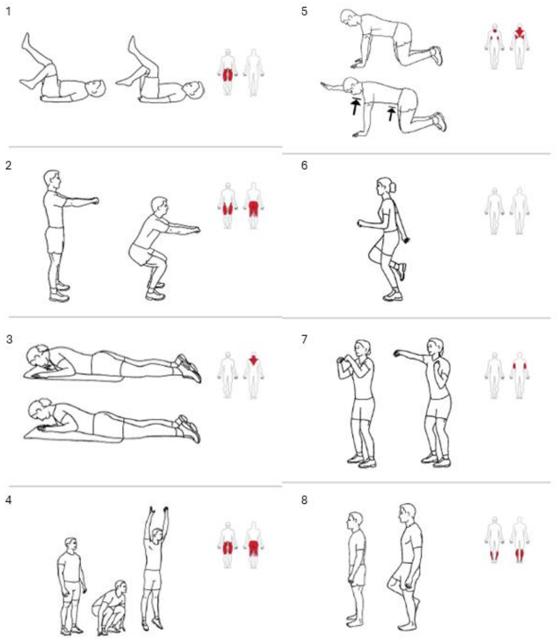

MEDIUM

1. Supine Leg Cycling

Lie on your back with your arms by your sides. Lift both legs off the floor and cycle with the legs while keeping your lower back pressed into the mat.

2. Squats

Stand with your feet hip-width apart. Keep your back straight and gaze forward. Bend your knees until the thighs are parallel to the floor. Push back up to standing. Optionally hold the squat position for a few seconds before rising.

3. Back Extension with Arm Sweep

Lie on your stomach with hands outside the shoulders. Lift the upper body and perform a large swimming motion with the arms. Lower slowly back down.

4. Lying Down to Standing

From standing, bend the knees and lower yourself down to the floor. Return to standing using any method you prefer.

5. Quadruped Diagonal Lift

Stand on all fours. Lift the opposite arm and leg at the same time while keeping the body stable. Lower slowly and repeat in a diagonal pattern. Sets: ___ Reps: ___

6. Running in Place

Run in place with feet parallel. Roll through the foot and lift the heels toward the glutes.

7. Boxing

Stand with feet shoulder-width apart and arms in a boxing guard. Alternate punches forward with clenched fists.

8. Single-Leg Balance with Movement

Stand on one leg with hands on hips. Lift the opposite leg forward, then backward, then out to the side. Return carefully to start. Keep the torso and pelvis stable.

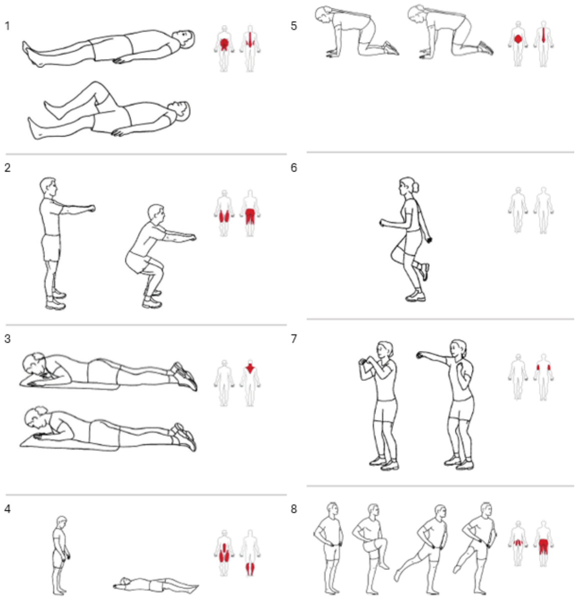

HARD

1. Diagonal Sit-Ups

Lie on your back with heels lifted off the floor. Bring one knee toward the opposite elbow, return slowly, and repeat on the other side.

2. Squats with Arms Overhead

Stand upright with feet hip-width apart and arms extended overhead. Squat to approximately 90 degrees while keeping knees aligned with toes, chest open, and gaze forward.

3. Back Extension with Straight Arms

Lie on your stomach with arms extended overhead. Engage the glutes and back muscles and lift the upper body and arms. Hold for 3–5 seconds, then lower and rest before repeating. Sets: ___ Reps: ___

4. Lying Down to Jump

From standing, bend the knees and place hands on the floor. Roll backward to a supine position with arms overhead. Stand up and perform a vertical jump, clapping hands overhead with straight arms.

5. Running in Place

Run in place with feet parallel. Roll through the foot and lift heels toward the glutes.

6. Quadruped Diagonal Arm and Leg Lift

Stand on all fours with head aligned to the spine. Engage the core and alternate lifting opposite arm and leg. Keep pelvis and lower back stable throughout.

7. Boxing

Stand with feet shoulder-width apart and arms in boxing position. Alternate punches forward with clenched fists.

8. Single-Leg Stance with Arm and Leg Movements

Stand on one leg and move arms and legs diagonally. Focus on maintaining balance and control.

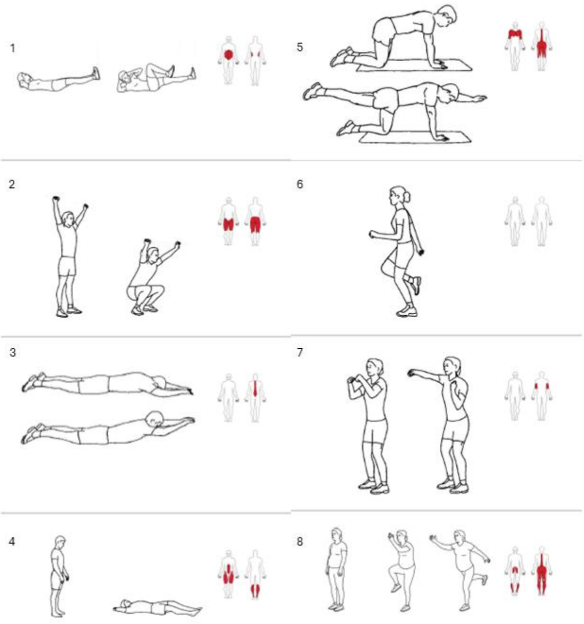

Illustrations created by Moa Almer in ExorLive (www.exorlive.com).

B) Relaxation Program: Yoga and Mindfulness Exercises

Instructions for the facilitators:

- Speak calmly and peacefully. Use pauses to allow for reflection and to increase the sense of calm.
- “A Journey Through the Body” can be done seated if someone doesn’t want to or cannot lie down.

Relaxation

We will now, over the next five minutes or so, do four relaxation exercises, which will end with you lying on your back on the floor. After the four exercises, I want you to stay lying on the floor, because we will do one final, longer relaxation exercise. I want you to focus on doing the exercises slowly—about as slowly as [Name] demonstrates them.

Exercise 1 – Tadasana (Mountain Pose) with Arm Movement; Standing Breathing Exercise – 6 repetitions

Start by letting your arms hang naturally at your sides. Slowly raise both arms toward the ceiling at the same time. When your hands reach above your head, let your palms touch. Bring your hands down toward your chest. Release and let your arms hang by your sides.

We will repeat the movement. Arms up toward the ceiling. Hands meet above your head. Bring them down toward your chest. Release, let arms hang.

Now, if you can, synchronize the movement with your breathing. Inhale as you raise your arms upward, exhale as you lower your hands. Let’s do it together:

- Lift your arms and inhale.
- Bring your hands down and exhale.
- Lift arms and inhale. Palms meet above your head.
- Exhale and lower your hands to your chest.

Again:

- Lift arms and inhale, palms meet, exhale, hands down.
- Release and let your arms hang.

One last time:

- Lift arms and inhale, palms meet, exhale and lower to the chest.
- Release and let arms hang.

Well done. We will now move on to the next exercise.

Exercise 2 – Tiriyaka Tadasana (Swaying Palm)

We will now do the second exercise. First, we will explain and show it, then we will do it together. If you feel confident, you can join in right from the start.

- Lift your arms toward the ceiling.
- Bend sideways as you exhale.
- Stretch back toward the ceiling and inhale.
- Bend to the other side as you exhale, then stretch and inhale.

Let’s do it together:

- Bend and exhale, stretch and inhale.
- Bend and exhale, stretch and inhale.
- One last time on each side: bend and exhale, stretch and inhale. Bend and exhale, stretch and inhale.

Lower your arms. Very good. We will now move on to the third exercise.

Exercise 3 – Marjaryasana/Bitilasana (Cat-Cow Pose)

Now we will do the third exercise. We will demonstrate it first. If you feel confident, you can join in from the beginning.

- Get on all fours, with your hands roughly under your shoulders and knees under your hips.
- Look up at the ceiling and arch your back, so your stomach moves toward the floor. Inhale as you do this.
- Now look slightly downward and backward toward the floor and round your back toward the ceiling. Exhale as you do this.

Well done. Now we will do it three times together:

- Look up, inhale, arch.
- Look down, exhale, round.
- Look up, inhale, arch.
- Look down, exhale, round.
- One last time: look up, inhale, arch. Look down, exhale, round.

Good. Now lie down on your back.

Exercise 4 – Apanasana (Knees-to-Chest Pose)

Lie on your back. Bend your legs toward your chest and hold your knees so your legs stay in place. You can either hold your knees with your hands or hug your legs with your arms—whichever feels best for you. Stay here for a moment. If you like, you can gently rock from side to side.

Now release your legs and stretch them out.
We will now move on to the final exercise.

Exercise 5 – "A Journey Through the Body"

Lie on your back as comfortably as you can. You may choose to keep your eyes open or closed. Relax your body as much as possible. Take three deep breaths:

- Inhale, exhale (x 3)

Now we will do an exercise called "A Journey Through the Body," and I will guide you:

- Start at your feet. Notice how your feet feel against the floor. Perhaps they feel heavy, or maybe your feet feel different from each other.
- Notice your heels, then your calves. Feel how your calves rest against the floor—maybe there's a little space beneath your lower legs.
- Move your attention to your knees and then your thighs. Feel how your thighs rest on the floor—maybe heavy, warm, or something else.
- Notice your hips and then your back. Perhaps there is a little space between your lower back and the floor. Feel your upper back and shoulders.
- Notice how your arms rest on the floor, your hands and fingers. Perhaps your body feels different on each side.
- Feel how your head rests on the floor. Relax your entire body.

Take five deep breaths:

- Inhale, exhale (x 5)

Stay lying down for a moment and rest until I signal [about a ten-second pause].

Now it's time to start moving again. Gently move your hands and feet, arms and legs. If you like, you can yawn or stretch. When you feel ready, open your eyes and sit up.

The relaxation is now complete. Well done.
